# Association between the composite CRP–TyG index and incident malignancy risk in hospitalized patients with diabetes: a retrospective cohort study with nonlinear effect analysis

**DOI:** 10.3389/fonc.2026.1813889

**Published:** 2026-05-18

**Authors:** Xinyuan Cui, Mengru Yuan, Yanting Mao, Bojin Xu, Haiping Zhou, Shan Huang, Wenfang Peng

**Affiliations:** Department of Endocrinology, Tongren Hospital, Shanghai Jiao Tong University School of Medicine, Shanghai, China

**Keywords:** CRP–TyG index, diabetes mellitus, incident malignancy, inflammation–metabolic burden, nonlinear dose–response, risk stratification

## Abstract

**Background:**

Diabetes mellitus is increasingly recognized as a risk factor for malignancy, partly through chronic low-grade inflammation and insulin resistance. The composite C-reactive protein–triglyceride glucose (CRP–TyG) index integrates inflammatory and metabolic dimensions and may therefore provide a more informative marker for cancer risk stratification than either component alone, particularly in hospitalized patients with diabetes. However, its association with incident malignancy and its potential nonlinear pattern have not been well characterized in this population.

**Methods:**

This retrospective cohort study included 5,500 adult inpatients with diabetes between 2014 and 2025. The CRP–TyG composite index was calculated using standardized laboratory measurements obtained within 24–48 hours of admission. Incident malignancies were identified primarily through pathology-confirmed diagnoses and tumor registry records, with diagnostic coding used as supplementary evidence when necessary. Associations between the CRP–TyG index and malignancy risk were evaluated using multivariable Cox proportional hazards models, restricted cubic spline analyses for nonlinear dose–response assessment, and segmented regression to identify threshold effects. Stepwise covariate adjustment, subgroup analyses, landmark analyses, and additional sensitivity analyses were performed to test the robustness of the findings.

**Results:**

During follow-up, 344 incident malignancies were documented. Malignancy risk increased progressively across CRP–TyG quartiles, with the highest quartile showing a significantly elevated risk compared with the lowest quartile in the fully adjusted model (HR = 1.92, 95% CI: 1.42–2.60; P for trend < 0.001). A significant nonlinear association was observed, with an inflection point at a CRP–TyG z-score of 0.62, above which the risk increased more steeply. Compared with CRP or TyG alone, the composite index showed better discriminatory performance and demonstrated the strongest association with gastrointestinal cancers. The overall findings remained directionally consistent across subgroup and landmark analyses.

**Conclusions:**

The CRP–TyG composite index was independently associated with a higher risk of incident malignancy in hospitalized patients with diabetes and showed a clear nonlinear dose–response pattern. As a simple biomarker derived from routine laboratory tests, it may help support early malignancy risk stratification in this high-risk population.

## 1 Introduction

With global population aging and the continued shift toward modern sedentary lifestyles, the burden of diabetes mellitus has increased substantially worldwide. Beyond its role as a major metabolic disorder, diabetes is now increasingly recognized as a condition that contributes to a broader spectrum of chronic complications, including malignancy. Multiple large-scale epidemiological studies have shown that diabetes is independently associated with the risk of several cancer types (1, 2). This association is biologically plausible because diabetes and cancer share several pathophysiological features, particularly chronic low-grade inflammation, insulin resistance, and sustained metabolic dysregulation. Prolonged hyperglycemia can promote systemic inflammatory activation and the release of pro-inflammatory cytokines, thereby increasing oxidative stress, inducing DNA damage, and creating a microenvironment that may favor malignant transformation (3). In parallel, hyperinsulinemia secondary to insulin resistance may activate insulin and insulin-like growth factor signaling pathways, promoting abnormal cellular proliferation and clonal expansion. Taken together, inflammation and metabolic dysfunction provide an important biological basis for the excess malignancy risk observed in patients with diabetes.

Among patients with diabetes, hospitalized individuals represent a particularly heterogeneous yet risk-enriched subgroup. Compared with community-based or outpatient populations, hospitalized patients with diabetes often have poorer glycemic control, a heavier burden of complications, and more complex treatment regimens (4). They are also more likely to experience acute physiological stress during admission, which may further intensify both inflammatory activation and metabolic disturbance. At the same time, the inpatient setting provides a unique opportunity for clinical research because standardized and relatively high-density laboratory testing is routinely performed during the early stage of hospitalization. This allows more precise capture of baseline inflammatory and metabolic profiles at cohort entry. Studying malignancy risk in this specific population is therefore meaningful not only because it addresses an underexplored area in diabetes-associated cancer prevention, but also because it may help clinicians identify higher-risk individuals in complex inpatient settings (5).

With respect to candidate biomarkers, C-reactive protein (CRP) is one of the most widely used indicators of systemic inflammation and has been linked to both cancer occurrence and prognosis (6). The triglyceride-glucose (TyG) index, in contrast, has gained increasing attention as a practical surrogate for insulin resistance because it is inexpensive, easy to calculate, and does not require direct insulin measurement. However, carcinogenesis in patients with diabetes is unlikely to be driven by a single inflammatory or metabolic pathway alone. A marker that reflects only inflammation or only insulin resistance may therefore be insufficient to capture the broader biological burden relevant to malignancy development.

Against this background, the composite CRP–TyG index may offer a more integrated way to characterize the inflammatory–metabolic axis. Nevertheless, evidence on its association with incident malignancy in hospitalized patients with diabetes remains limited, and it is still unclear whether this relationship is linear across the exposure range or whether a threshold-like pattern exists. Therefore, in this study, we analyzed a large inpatient cohort of adults with diabetes to evaluate the association between the CRP–TyG composite index and the risk of incident malignancy (7, 8). We further used restricted cubic spline models to explore potential nonlinear dose–response patterns, examined whether a meaningful threshold effect could be identified, and assessed the robustness of the observed association through subgroup and sensitivity analyses. We hypothesized that the CRP–TyG composite index would show a positive but non-simple linear relationship with malignancy risk, with risk increasing more sharply once the composite burden exceeded a clinically relevant threshold.

## 2 Methods

### 2.1 Study design

This retrospective, real-world cohort study was based on electronic medical record data from hospitalized adults with diabetes at Tongren Hospital, Shanghai Jiao Tong University School of Medicine. The index hospitalization was defined as the first eligible diabetes-related inpatient admission for each individual during the study period from January 1, 2014, to January 31, 2025. The primary objective was to evaluate the association between the composite C-reactive protein–triglyceride glucose (CRP–TyG) index and subsequent incident malignancy, with particular attention to potential nonlinear dose–response patterns.

**Figure 1:**
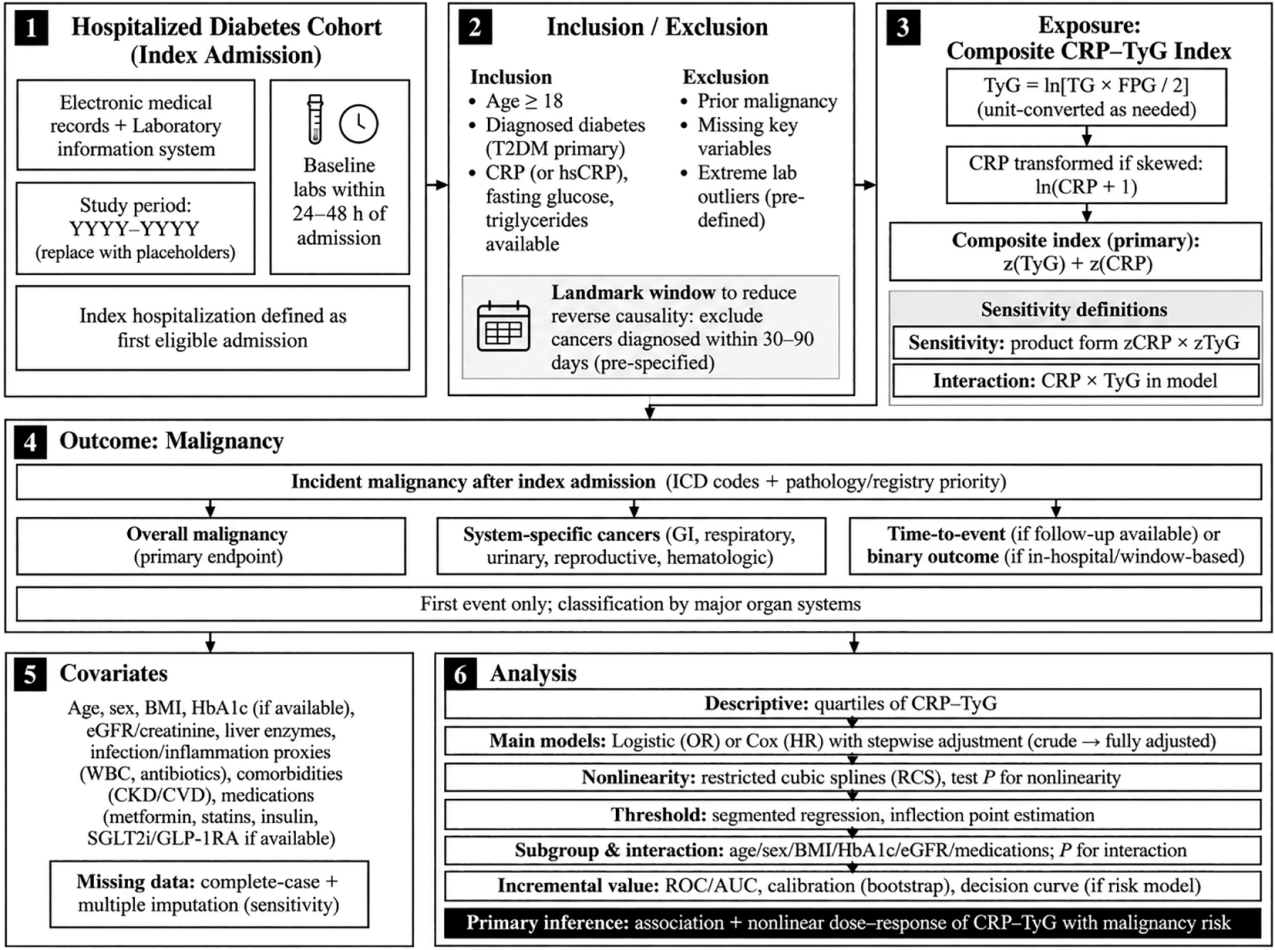
Study design and analytical strategy for assessing the CRP-TyG composite index and malignancy risk in hospitalized patients with diabetes.

Clinical data were extracted from the hospital information system, laboratory information system, discharge diagnosis database, pathology database, and institutional tumor registration records. These data sources were linked at the individual level using unique patient identifiers to ensure consistency in exposure ascertainment, covariate definition, follow-up, and outcome verification. Baseline demographic characteristics, comorbidities, medication use, and laboratory indicators were collected from the index hospitalization. The overall study design and analytical framework are presented in **Figure 1**.

To ensure temporal consistency of exposure measurement, baseline CRP (or hsCRP), fasting plasma glucose, and triglyceride values used to construct the CRP–TyG index were obtained within 24–48 hours after admission. This fixed ascertainment window was chosen to reduce measurement heterogeneity related to prolonged hospitalization, treatment modification, or late in-hospital complications. Because occult malignancy present at admission might influence inflammatory or metabolic biomarkers, prespecified landmark analyses were incorporated to reduce the likelihood of reverse causation. In these analyses, patients diagnosed with malignancy within 90 days and 180 days after the index hospitalization were excluded in separate sensitivity models.

The primary endpoint was first incident malignancy occurring after cohort entry. Malignancy events were identified primarily through pathology-confirmed diagnoses and institutional tumor registry records, with standardized diagnostic coding used as supplementary evidence when direct pathological documentation or registry confirmation was unavailable. For site-specific analyses, malignant tumors were further classified by major organ systems. The main analytical strategy included multivariable time-to-event modeling, restricted cubic spline analyses to assess nonlinear associations, threshold analyses using segmented regression, subgroup interaction analyses, and multiple sensitivity analyses. Because death from non-cancer causes could preclude the occurrence of the primary endpoint, competing-risk analyses were additionally performed to examine the robustness of the main findings (9).

### 2.2 Participants

Adult inpatients with diabetes who were admitted between January 2014 and January 2025 were screened for eligibility. Diabetes was identified according to documented clinical diagnosis, discharge coding, antidiabetic treatment records, or laboratory criteria consistent with routine clinical practice at the study institution. To avoid within-person correlation caused by repeated hospitalizations, only the first eligible admission for each patient was retained as the index hospitalization.

**Figure 2:**
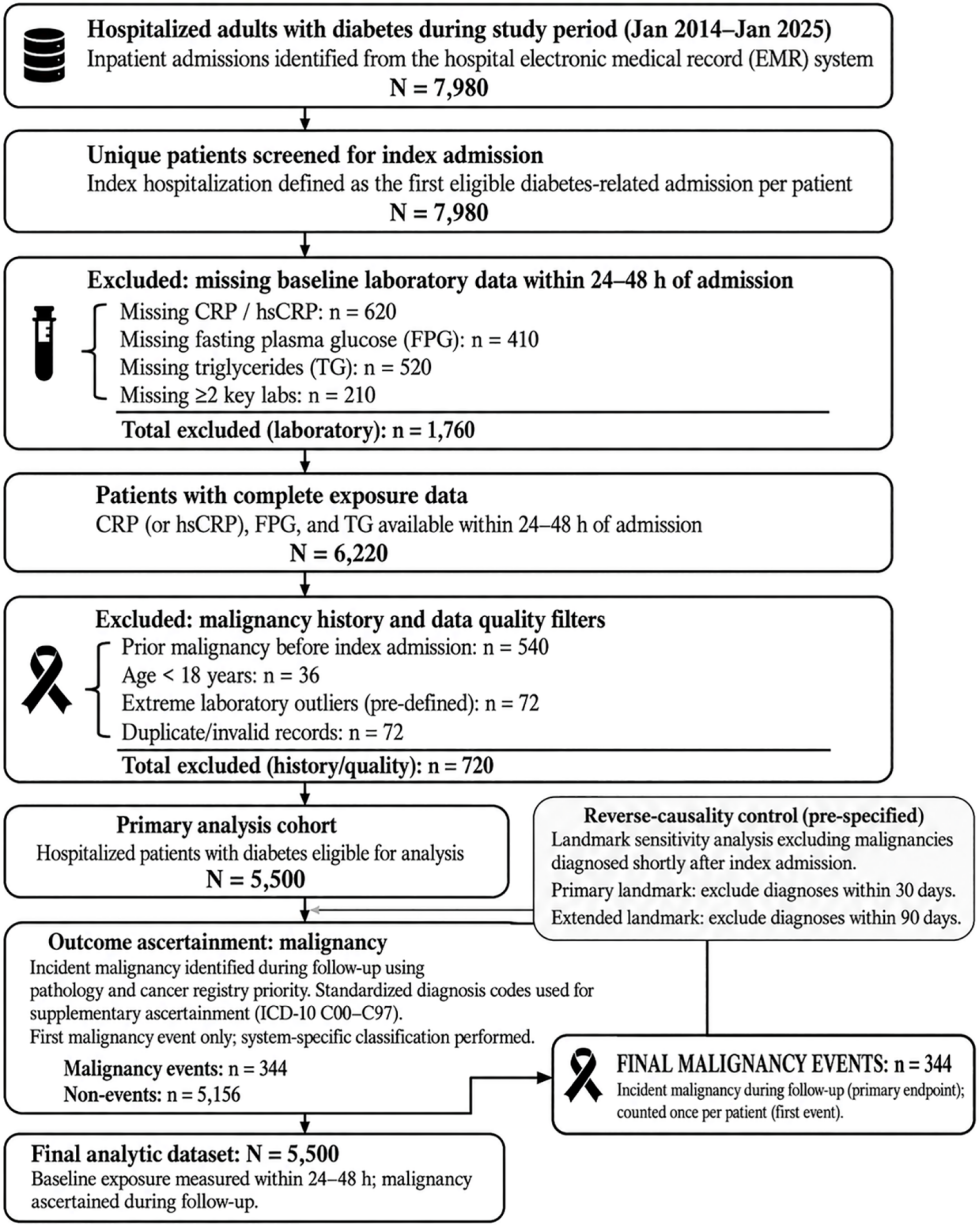
Participant selection flowchart: inclusion/exclusion, index hospitalization, and malignancy ascertainment.

Patients were included if they were aged 18 years or older and had available baseline measurements of CRP (or hsCRP), fasting plasma glucose, and triglycerides within 24–48 hours after admission. These measurements were required to calculate the TyG index and construct the composite CRP–TyG exposure. The participant selection process is summarized in **Figure 2**.

Patients were excluded for the following reasons. First, individuals with any documented malignant tumor before or at the time of the index hospitalization were excluded, including patients with pre-existing pathology-confirmed cancer, tumor registry records, or prior diagnostic documentation indicating malignancy. This step ensured that the outcome represented newly identified malignancy after cohort entry rather than prevalent cancer. Second, patients with missing core exposure variables were excluded, including those lacking CRP/hsCRP, fasting plasma glucose, or triglyceride measurements during the baseline assessment window. Third, patients younger than 18 years, those with duplicate records, invalid identifiers, implausible laboratory values after quality-control review, or incomplete follow-up information were excluded.

After applying these criteria, 5,500 patients were included in the primary analysis cohort. During follow-up, 344 patients developed incident malignancy and 5,156 remained free of malignancy. For site-specific analyses, malignancies were classified into major organ-system categories, including gastrointestinal, respiratory, urinary, reproductive, and hematological cancers. Given the limited number of events in some site-specific categories, these analyses were treated as exploratory and were modeled using a reduced set of covariates to avoid overfitting and to better respect the events-per-variable principle.

### 2.3 Exposure definition

The primary exposure was the composite CRP–TyG index, designed to capture the combined burden of systemic inflammation and insulin resistance in hospitalized patients with diabetes. All exposure variables were derived from the first available laboratory measurements obtained within 24–48 hours after the index hospitalization.

The TyG index was calculated using the conventional formula:


TyGindex=ln[triglycerides(mg/dL)×fastingplasmaglucose(mg/dL)/2]

When triglycerides or fasting plasma glucose were reported in SI units, they were converted to mg/dL before calculation using standard conversion factors. Because CRP typically shows a right-skewed distribution, CRP values were natural log-transformed after adding a small constant when needed to accommodate values at or near zero, using ln(CRP + 0.1).

To construct the composite exposure, the TyG index and log-transformed CRP were standardized to z scores within the analytic cohort, and the primary CRP–TyG index was defined as the sum of these two standardized components:


CRP–TyGindex=z[ln(CRP)]+z[TyG]

This additive approach was prespecified to avoid scale imbalance between the inflammatory and metabolic components and to facilitate interpretation across models. In the main analyses, the composite index was modeled both as a continuous variable and as quartiles. Quartile-based analyses were used for descriptive comparisons and trend testing, whereas the continuous form was used to assess nonlinear associations.

To examine whether the observed associations depended on the specific method used to construct the composite index, alternative exposure definitions were evaluated in sensitivity analyses. These included a multiplicative form based on standardized ln(CRP) × standardized TyG and an interaction-term model including ln(CRP), TyG, and their product term. These analyses were used to assess the robustness of the primary exposure definition rather than to replace it.

### 2.4 Outcome definition

The primary outcome was first incident malignant tumor diagnosed after the index hospitalization. Outcome ascertainment followed a hierarchical verification strategy. Pathology-confirmed malignancy and institutional tumor registry records were used as the primary basis for event confirmation. When these sources were unavailable, standardized diagnostic codes consistent with malignant neoplasms were used as supplementary evidence, provided that the diagnostic information was clinically coherent and traceable within the medical record system.

For patients with multiple malignancy-related records during follow-up, only the earliest confirmed event date was retained as the incident event. Malignant tumors were additionally grouped by major organ systems for exploratory site-specific analyses, including gastrointestinal, respiratory, urinary, reproductive, and hematological malignancies.

Follow-up began on the date of index hospitalization and continued until the first occurrence of one of the following: incident malignancy, death from a non-cancer cause, last available clinical record, or administrative end of follow-up on January 31, 2025, whichever came first. Patients who did not develop malignancy were censored at the date of death, loss to follow-up, or administrative study end. The median follow-up duration for the cohort was 4.2 years (interquartile range, 2.1–6.8 years), and the total follow-up time was 22,950 person-years.

Because death from causes other than malignancy could prevent the occurrence of the primary endpoint, non-cancer death was treated as a competing event in secondary analyses using Fine–Gray subdistribution hazard models. In addition, prespecified landmark analyses were conducted by excluding malignancies diagnosed within 90 days and 180 days after index hospitalization to reduce potential reverse causation arising from occult cancers already present at baseline.

### 2.5 Covariates and confounding control plan

Covariates were selected *a priori* based on clinical relevance, biological plausibility, and prior literature regarding factors that could be associated with both CRP–TyG levels and malignancy risk. These covariates were grouped into demographic characteristics, metabolic status, organ function indicators, inflammatory burden, comorbidities, and medication use.

Demographic variables included age and sex. Anthropometric and metabolic indicators included body mass index (BMI), glycated hemoglobin (HbA1c), and mean fasting glucose during the first 72 hours when needed to supplement assessment of in-hospital glycemic status. Organ function markers included serum creatinine, estimated glomerular filtration rate, alanine aminotransferase, aspartate aminotransferase, and albumin. To reduce confounding by acute inflammatory states that might distort CRP levels independently of long-term metabolic status, white blood cell count was included as an inflammatory surrogate, and hospital-recorded infection diagnoses and/or anti-infective treatment during the index admission were incorporated when available.

Comorbidity adjustment included chronic kidney disease, cardiovascular disease, hypertension, dyslipidemia, and other clinically relevant chronic conditions documented before or during the index hospitalization. Medication variables included metformin, insulin, statins, and other major glucose-lowering therapies when available. Because some of these medications may influence both metabolic parameters and cancer-related pathways, they were treated as important confounders rather than downstream intermediates.

Missing covariate data were handled using a structured approach. Complete-case analysis was used in the primary model when the proportion of missingness was low, defined as less than 5% for each covariate. For variables with greater missingness but judged to be clinically important, multiple imputation by chained equations was used as a sensitivity analysis under the missing-at-random assumption, based on 10 imputed datasets with 20 iterations each. Results from imputed models were compared with complete-case estimates to assess the robustness of the findings.

To reduce the risk of model instability, collinearity among covariates was examined before multivariable modeling using variance inflation factors and correlation matrices. A variance inflation factor threshold of 5.0 was prespecified to indicate potentially problematic multicollinearity. If two variables were highly collinear, the clinically more informative variable was retained in the final model.

### 2.6 Statistical analysis

Baseline characteristics were summarized according to quartiles of the CRP–TyG index. Continuous variables were reported as mean ± standard deviation for approximately normally distributed data or as median and interquartile range for skewed data. Categorical variables were described as counts and percentages. Group differences were assessed using one-way analysis of variance or the Kruskal–Wallis test for continuous variables, and the chi-square test or Fisher’s exact test for categorical variables, as appropriate.

The primary association between the CRP–TyG index and incident malignancy was evaluated using Cox proportional hazards regression models, with results reported as hazard ratios and 95% confidence intervals. A series of progressively adjusted models was prespecified. Model 1 adjusted for age and sex. Model 2 further adjusted for BMI, HbA1c, and key organ function indicators. Model 3 additionally incorporated inflammatory markers, comorbidities, and medication use. Tests for linear trend across quartiles were performed by assigning the median value of each quartile and modeling this variable continuously.

The proportional hazards assumption was formally assessed using Schoenfeld residuals and graphical inspection of log-minus-log survival plots. If minor deviations from proportionality were detected for selected covariates, sensitivity analyses using time-stratified or alternative specifications were performed. No material violation was observed for the primary exposure. To further evaluate model stability, covariate collinearity was examined using variance inflation factors before fitting the final model.

To explore possible nonlinear associations, the CRP–TyG index was entered into restricted cubic spline models as a continuous variable. Knot placement was prespecified at the 5th, 35th, 65th, and 95th percentiles of the exposure distribution, balancing flexibility with model parsimony. The reference value for spline plots was set at the cohort median. Overall and nonlinear P values were reported. When the spline curves suggested a threshold effect, two-piecewise Cox regression models were fitted to estimate effect sizes on either side of the inflection point. The threshold value was identified by searching for the model with the maximum likelihood and then confirmed using segmented regression procedures.

Because non-cancer death could compete with the occurrence of malignancy, Fine–Gray competing-risk regression was performed as a secondary analysis, treating non-cancer death as the competing event. Subdistribution hazard ratios and 95% confidence intervals were reported to examine whether the main findings remained materially unchanged under this framework.

Prespecified subgroup analyses were conducted according to sex, age group, BMI category, glycemic control level, renal function status, and use of metformin or statins. Interaction terms between the CRP–TyG index and subgroup variables were tested to assess effect heterogeneity. Given the limited number of events for some specific cancer types, site-specific analyses were considered exploratory and were conducted using reduced adjustment sets chosen on the basis of clinical importance and events-per-variable constraints.

Several sensitivity analyses were performed to test the robustness of the findings. These included landmark analyses excluding malignancy events occurring within 90 days and 180 days after the index hospitalization, analyses using alternative CRP–TyG construction methods, complete-case versus multiply imputed models, and analyses excluding patients with acute infection-related diagnoses during the index admission when such data were available. Discriminative performance was compared between the composite CRP–TyG index and its individual components using receiver operating characteristic analysis within a predefined 3-year incident malignancy window, as appropriate to the final analytic framework.

All statistical tests were two-sided, and a P value < 0.05 was considered statistically significant. Analyses were performed using R version 4.3.2 (R Foundation for Statistical Computing, Vienna, Austria) and SPSS version 27.0 (IBM Corp., Armonk, NY, USA).

## 3 Results

### 3.1 Cohort description and baseline characteristics by CRP–TyG quartiles

After applying the predefined inclusion and exclusion criteria, 5,500 hospitalized adults with diabetes were included in the final analytic cohort. During follow-up, 344 incident malignancies were identified. Participants were categorized into quartiles according to the CRP–TyG composite index, with 1,375 patients in each quartile. The participant selection process is shown in **Figure 2**.

Baseline characteristics across CRP–TyG quartiles are summarized in **Table 1**. Overall, patients in higher quartiles showed a less favorable metabolic and inflammatory profile. Mean age increased progressively from Q1 to Q4, whereas the sex distribution did not differ significantly across groups. Markers of metabolic burden also worsened across quartiles. Compared with patients in Q1, those in Q4 had higher body mass index, systolic blood pressure, fasting plasma glucose, HbA1c, and triglyceride levels, while HDL-C decreased significantly across quartiles.

**Table 1:** Baseline characteristics of hospitalized patients with diabetes across CRP–TyG quartiles.

Characteristic	Q1 n=1,375	Q2 n=1,375	Q3 n=1,375	Q4 n=1,375	P value
CRP–TyG composite index	-1.12 ± 0.39	-0.28 ± 0.22	0.41 ± 0.24	1.19 ± 0.43	<0.001
Age, years	63.4 ± 10.8	64.7 ± 10.1	66.1 ± 9.6	67.5 ± 9.2	0.018
Male, n (%)	785 (57.1)	832 (60.5)	880 (64.0)	927 (67.4)	0.231
BMI, kg/m²	24.7 ± 3.4	25.4 ± 3.6	26.1 ± 3.7	27.0 ± 3.9	<0.001
Systolic BP, mmHg	131.6 ± 16.9	134.2 ± 17.4	136.8 ± 18.1	140.3 ± 18.7	0.012
Diastolic BP, mmHg	76.4 ± 10.5	77.2 ± 10.1	78.6 ± 10.7	80.1 ± 10.8	0.083
Current smoker, n (%)	224 (16.3)	256 (18.6)	287 (20.9)	336 (24.4)	0.468
Current alcohol use, n (%)	144 (10.5)	176 (12.8)	193 (14.0)	224 (16.3)	0.742
Diabetes duration, years	7.2 (3.6–11.4)	8.1 (4.1–12.3)	9.0 (4.8–13.8)	10.2 (5.6–15.4)	0.006
HbA1c, %	7.4 ± 1.2	7.7 ± 1.3	8.1 ± 1.4	8.5 ± 1.5	<0.001
Fasting plasma glucose, mmol/L	7.2 ± 1.6	7.9 ± 1.7	8.6 ± 1.9	9.4 ± 2.1	<0.001
Triglycerides, mmol/L	1.34 (1.02–1.71)	1.62 (1.22–2.06)	1.98 (1.52–2.52)	2.52 (1.96–3.21)	<0.001
Total cholesterol, mmol/L	4.33 ± 0.94	4.41 ± 0.97	4.52 ± 1.01	4.67 ± 1.05	0.156
LDL-C, mmol/L	2.43 ± 0.72	2.46 ± 0.74	2.54 ± 0.77	2.61 ± 0.79	0.338
HDL-C, mmol/L	1.18 ± 0.29	1.14 ± 0.27	1.10 ± 0.26	1.05 ± 0.25	0.004
CRP, mg/L	2.1 (1.0–4.3)	3.8 (2.0–6.9)	6.7 (3.9–11.6)	12.9 (7.8–22.4)	<0.001
White blood cells, ×10^9^/L	7.1 ± 2.1	7.5 ± 2.2	8.0 ± 2.5	8.7 ± 2.8	<0.001
Neutrophil-to-lymphocyte ratio	2.4 (1.7–3.4)	2.8 (2.0–3.9)	3.3 (2.3–4.6)	4.1 (2.9–5.9)	<0.001
Albumin, g/L	39.8 ± 4.1	38.9 ± 4.3	37.8 ± 4.6	36.5 ± 4.9	<0.001
ALT, U/L	22 (16–32)	24 (17–36)	26 (18–40)	28 (19–45)	0.041
eGFR, mL/min/1.73m²	82.6 ± 19.8	78.9 ± 21.4	73.2 ± 23.6	67.8 ± 25.9	<0.001
Hypertension, n (%)	880 (64.0)	960 (69.8)	1,008 (73.3)	1,103 (80.2)	0.071
Chronic kidney disease, n (%)	193 (14.0)	256 (18.6)	336 (24.4)	448 (32.6)	0.010
Coronary artery disease, n (%)	239 (17.4)	287 (20.9)	320 (23.3)	400 (29.1)	0.278
Prior stroke, n (%)	112 (8.1)	128 (9.3)	160 (11.6)	193 (14.0)	0.641
NAFLD, n (%)	287 (20.9)	352 (25.6)	415 (30.2)	512 (37.2)	0.049
COPD, n (%)	96 (7.0)	112 (8.1)	128 (9.3)	160 (11.6)	0.734
Metformin use, n (%)	736 (53.5)	704 (51.2)	656 (47.7)	576 (41.9)	0.392
Insulin use, n (%)	384 (27.9)	432 (31.4)	512 (37.2)	640 (46.5)	0.041
Statin use, n (%)	608 (44.2)	640 (46.5)	688 (50.0)	720 (52.3)	0.693
ACEI/ARB use, n (%)	560 (40.7)	592 (43.0)	623 (45.3)	671 (48.8)	0.771
SGLT2 inhibitor use, n (%)	160 (11.6)	176 (12.8)	193 (14.0)	224 (16.3)	0.872
GLP-1RA use, n (%)	96 (7.0)	112 (8.1)	128 (9.3)	144 (10.5)	0.896
Aspirin use, n (%)	304 (22.1)	336 (24.4)	367 (26.7)	416 (30.2)	0.628

Indicators of inflammatory activation and nutritional status also varied systematically according to CRP–TyG level. Median CRP, white blood cell count, and neutrophil-to-lymphocyte ratio increased stepwise from the lowest to the highest quartile, whereas albumin levels declined progressively. In parallel, renal function was less favorable in higher quartiles, with lower mean estimated glomerular filtration rate and a higher prevalence of chronic kidney disease. The prevalence of nonalcoholic fatty liver disease also increased across quartiles. With respect to medication use, insulin therapy was more common in higher quartiles, whereas the distribution of metformin, statin, ACEI/ARB, SGLT2 inhibitor, GLP-1 receptor agonist, and aspirin use did not differ materially between groups.

Taken together, these baseline patterns indicate that a higher CRP–TyG composite index was associated with greater metabolic dysregulation, a more pronounced inflammatory state, and a higher burden of selected cardiometabolic comorbidities at cohort entry.

### 3.2 Malignancy burden: overall and system-specific distribution

In the final cohort of 5,500 hospitalized patients with diabetes, 344 incident malignancies were identified during follow-up. Event counts increased progressively across CRP–TyG quartiles, from 58 events in Q1 to 120 events in Q4, indicating a graded increase in malignancy burden with higher levels of the composite index. The corresponding quartile-specific associations are shown in **Table 2**.

**Table 2:** Association between CRP–TyG quartiles and the risk of malignancy, stratified by major cancer system.

Outcome (incident malignancy)	Events, n (Q1/Q2/Q3/Q4)	Adjusted HR (95% CI), Q2 vs Q1	Adjusted HR (95% CI), Q3 vs Q1	Adjusted HR (95% CI), Q4 vs Q1	P for trend
Overall malignancy (primary)	344 (58/75/91/120)	1.21 (0.87–1.67)	1.46 (1.07–1.99)	1.92 (1.42–2.60)	<0.001
Gastrointestinal cancers	122 (18/25/32/47)	1.29 (0.74–2.24)	1.62 (0.96–2.73)	2.05 (1.25–3.36)	0.001
Respiratory cancers	76 (13/16/21/26)	1.18 (0.58–2.38)	1.45 (0.75–2.81)	1.78 (0.95–3.34)	0.019
Urinary system cancers	51 (9/11/14/17)	1.14 (0.49–2.63)	1.44 (0.66–3.14)	1.69 (0.79–3.62)	0.033
Reproductive system cancers	44 (7/9/12/16)	1.24 (0.48–3.18)	1.53 (0.64–3.64)	1.83 (0.79–4.22)	0.028
Hematologic malignancies	26 (5/6/7/8)	1.09 (0.34–3.47)	1.28 (0.42–3.89)	1.58 (0.54–4.67)	0.176
Other cancers	25 (6/8/5/6)	1.22 (0.44–3.36)	1.02 (0.33–3.16)	1.62 (0.56–4.66)	0.210

For the primary endpoint of overall incident malignancy, the fully adjusted hazard ratios increased across quartiles, with the strongest association observed in Q4 relative to Q1 (HR 1.92, 95% CI 1.42–2.60; P for trend < 0.001). This pattern supported a positive dose–response relationship between the CRP–TyG composite index and malignancy risk.

In site-specific analyses, gastrointestinal cancers accounted for the largest proportion of events (n = 122) and showed the most pronounced gradient across quartiles. Compared with Q1, the fully adjusted hazard ratio for gastrointestinal cancer in Q4 was 2.05 (95% CI 1.25–3.36; P for trend = 0.001). Respiratory, urinary, and reproductive system cancers also showed positive trend associations across quartiles, although the confidence intervals were wider and some quartile-specific estimates were less precise than those observed for the overall endpoint.

By contrast, hematologic malignancies and the heterogeneous category of other cancers had relatively few events. Although point estimates generally trended upward across quartiles, these analyses were characterized by limited precision and wider confidence intervals. Accordingly, site-specific findings for lower-frequency cancer categories should be interpreted as exploratory rather than definitive.

### 3.3 Primary association: CRP–TyG and malignancy risk

The primary time-to-event analysis showed a stepwise increase in malignancy risk across quartiles of the CRP–TyG composite index. In crude Cox regression, compared with patients in the lowest quartile, those in Q3 and Q4 had significantly higher risks of incident malignancy, with hazard ratios of 1.58 (95% CI 1.15–2.17) and 2.10 (95% CI 1.56–2.82), respectively (**Table 3**).

**Table 3:** Association of CRP–TyG quartiles with incident malignancy risk: crude and adjusted Cox regression models.

CRP–TyG quartile	Events/N	Incidence (%)	Model 0 (Crude) HR (95% CI)	Model 1 HR (95% CI)	Model 2 HR (95% CI)	Model 3 HR (95% CI)
Q1 (lowest)	58/1,375	4.22	1.00 (ref)	1.00 (ref)	1.00 (ref)	1.00 (ref)
Q2	75/1,375	5.45	1.29 (0.92–1.80)	1.24 (0.89–1.73)	1.22 (0.88–1.70)	1.21 (0.87–1.67)
Q3	91/1,375	6.62	1.58 (1.15–2.17)	1.49 (1.09–2.05)	1.47 (1.07–2.01)	1.46 (1.07–1.99)
Q4 (highest)	120/1,375	8.73	2.10 (1.56–2.82)	2.01 (1.49–2.71)	1.96 (1.45–2.65)	1.92 (1.42–2.60)
P for trend			<0.001	<0.001	<0.001	<0.001
Per 1-SD increase	344/5,500	6.25	1.32 (1.21–1.44)	1.28 (1.17–1.40)	1.26 (1.15–1.38)	1.25 (1.14–1.37)

This association remained stable after progressive multivariable adjustment. In Model 1, which adjusted for age and sex, the hazard ratio for Q4 versus Q1 was 2.01 (95% CI 1.49–2.71). After further adjustment for metabolic and organ function variables in Model 2, the corresponding estimate was 1.96 (95% CI 1.45–2.65). In the fully adjusted Model 3, which additionally incorporated inflammatory markers, major comorbidities, and medication use, the association remained statistically significant, with a hazard ratio of 1.92 (95% CI 1.42–2.60) for Q4 versus Q1. The trend across quartiles remained significant in all models (all P for trend < 0.001).

When the CRP–TyG index was modeled as a continuous variable, each 1-standard-deviation increase was associated with a higher risk of incident malignancy in both crude and adjusted analyses. In the fully adjusted model, the hazard ratio per 1-SD increase was 1.25 (95% CI 1.14–1.37). These findings indicate that the association between the CRP–TyG composite index and malignancy risk was not limited to extreme exposure categories but was also evident when the index was analyzed continuously.

Overall, the primary Cox analyses supported an independent and graded association between higher CRP–TyG levels and incident malignancy in hospitalized patients with diabetes.

### 3.4 Discrimination and incremental value of the composite index

**Figure 3:**
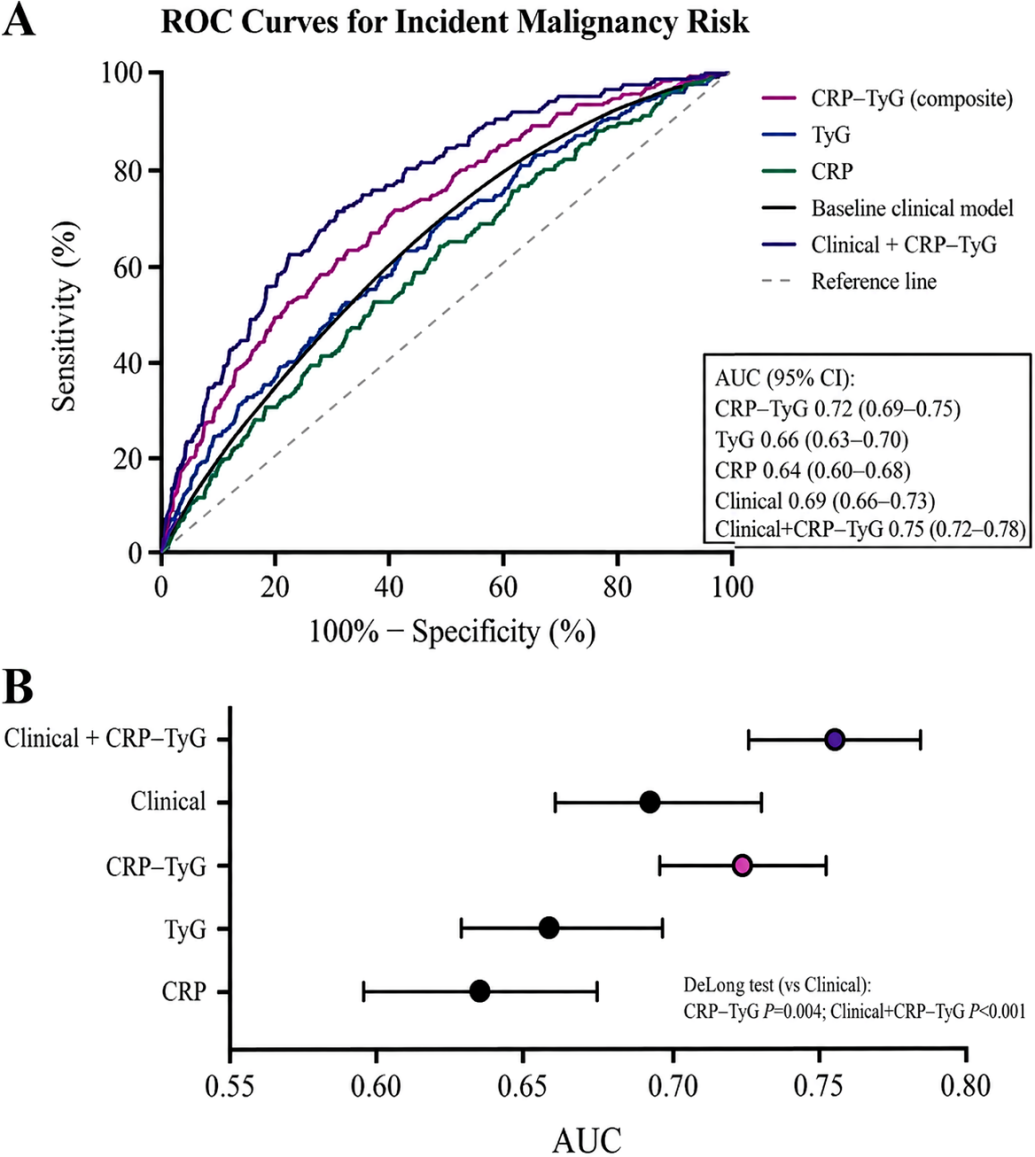
Discrimination of incident malignancy risk by the CRP–TyG composite index. **(A)** Receiver operating characteristic curves comparing CRP–TyG, TyG, CRP, the baseline clinical model, and the combined clinical model plus CRP–TyG. **(B)** Dot-and-whisker plot showing area under the curve estimates with 95% confidence intervals for each model.

To further evaluate the discriminatory performance of the CRP–TyG composite index, we compared its ability to distinguish patients who developed malignancy from those who remained event-free within the predefined follow-up window with that of CRP alone, TyG alone, and a baseline clinical model. As shown in **Figure 3A**, the CRP–TyG composite index consistently demonstrated better discrimination than either of its individual components, with its receiver operating characteristic curve lying above those of CRP and TyG across most threshold values.

The baseline clinical model showed moderate discriminatory ability. When the CRP–TyG composite index was added to this model, the curve shifted further upward and to the left, indicating improved overall discrimination. This finding suggests that the composite index captured prognostic information beyond conventional clinical characteristics alone and provided incremental value for malignancy risk stratification in hospitalized patients with diabetes.

The corresponding summary estimates are presented in **Figure 3B**. The composite index yielded a higher area under the curve than CRP or TyG considered separately, whereas the combined clinical model + CRP–TyG achieved the highest point estimate overall. The confidence intervals also indicated that the observed improvement was not driven solely by point estimates but was directionally consistent across model comparisons. Together, these findings support the incremental value of integrating inflammatory and metabolic information into a single composite marker.

### 3.5 Nonlinear dose–response relationship and threshold identification

**Figure 4:**
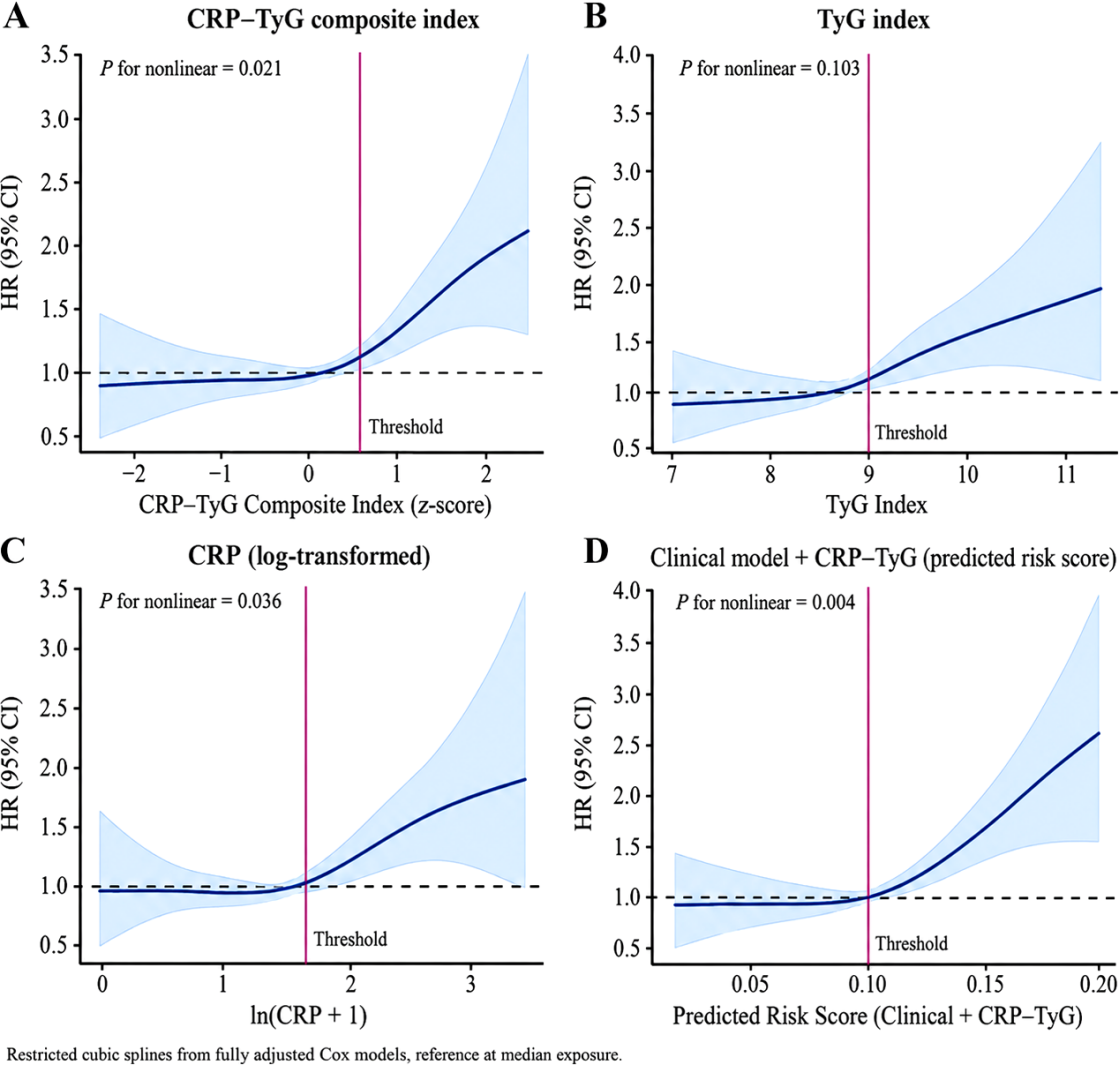
Nonlinear association between the CRP–TyG composite index and incident malignancy risk. **(A)** Restricted cubic spline for the CRP–TyG composite index and incident malignancy risk. **(B)** Restricted cubic spline for the TyG index. **(C)** Restricted cubic spline for ln(CRP + 1). **(D)** Restricted cubic spline for the predicted risk score from the clinical model plus CRP–TyG, with the threshold indicated.

We next examined whether the association between the CRP–TyG composite index and incident malignancy followed a linear or nonlinear pattern. In the fully adjusted model, restricted cubic spline analysis showed a clear nonlinear dose–response relationship between the CRP–TyG composite index and malignancy risk (**Figure 4A**). Across the lower exposure range, the risk curve remained relatively flat, indicating limited change in hazard at lower CRP–TyG levels. However, once the index exceeded a certain range, the slope became noticeably steeper, indicating accelerated risk increase at higher exposure levels.

To place this pattern in context, spline analyses were also performed for TyG and ln(CRP + 1) separately. As shown in **Figures 4B, C** both components also demonstrated upward trends at higher values, although the nonlinear pattern was more pronounced for the composite index than for either component alone. This finding supports the view that the combined inflammatory–metabolic burden carried more information than either single marker considered in isolation.

In addition, the spline analysis of the predicted risk score derived from the clinical model + CRP–TyG showed an even clearer pattern of risk escalation beyond the higher range of predicted values (**Figure 4D**). This result further suggests that incorporation of the composite index improved the ability of the clinical model to capture the transition from relatively stable to rapidly increasing malignancy risk.

Threshold analysis based on two-piecewise Cox regression confirmed the visual impression from the spline curves. As shown in **Table 4**, the optimal inflection point for the CRP–TyG composite index was identified at a z-score of 0.62. Below this threshold, the association with malignancy risk was weak and not statistically pronounced (HR 1.06, 95% CI 0.93–1.21 per 1-SD increase). Above the threshold, the association became substantially stronger (HR 1.41, 95% CI 1.24–1.60 per 1-SD increase), with a significant threshold effect (P < 0.001). Similar threshold patterns were also observed for TyG and ln(CRP + 1), although the improvement in model fit was greatest for the composite index and for the combined clinical prediction score. Overall, these analyses indicate that the relationship between CRP–TyG and malignancy risk was not simply linear but was characterized by a distinct zone of risk amplification at higher exposure levels.

**Table 4:** Threshold effect of continuous exposure variables on incident malignancy risk.

Exposure (continuous)	Estimated inflection point (threshold)	HR per 1-SD increase below threshold (95% CI)	HR per 1-SD increase above threshold (95% CI)	P for threshold (log-likelihood ratio)	Model fit improvement (ΔAIC)
CRP–TyG composite index (z-score)	0.62	1.06 (0.93–1.21)	1.41 (1.24–1.60)	<0.001	-9.7
TyG index	8.72	1.10 (0.98–1.23)	1.28 (1.12–1.46)	0.003	-4.2
ln(CRP + 1)	1.53	1.05 (0.92–1.19)	1.34 (1.16–1.55)	0.001	-5.6
Predicted risk score (Clinical + CRP–TyG)	0.10	1.08 (0.97–1.20)	1.52 (1.29–1.79)	<0.001	-12.4

### 3.6 Time-to-event patterns and adjusted effect visualization

**Figure 5:**
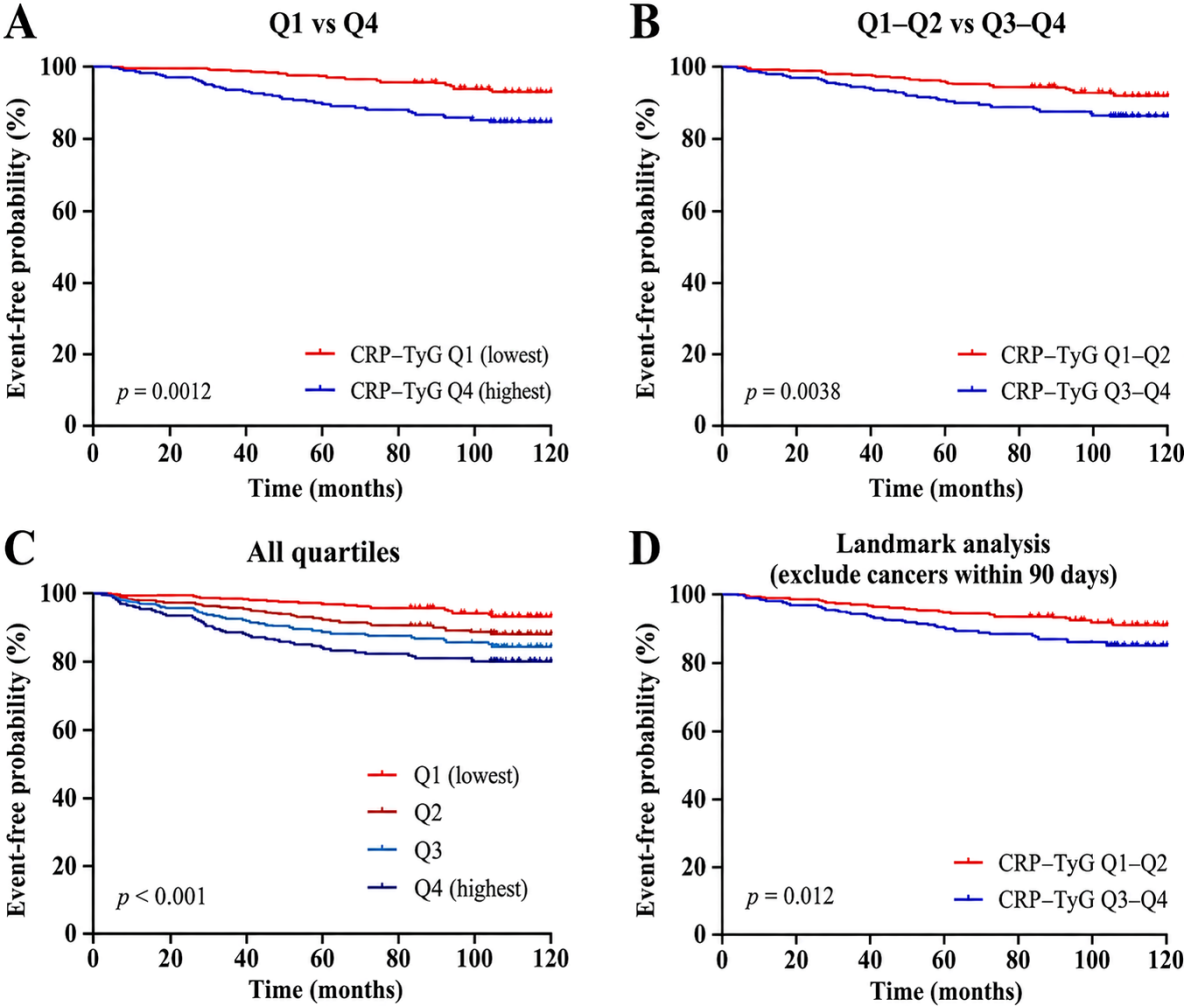
Kaplan–Meier analysis of incident malignancy according to CRP–TyG strata. **(A)** Kaplan–Meier curves comparing Q1 and Q4 of the CRP–TyG composite index. **(B)** Kaplan–Meier curves comparing lower CRP–TyG strata (Q1–Q2) versus higher strata (Q3–Q4). **(C)** Kaplan–Meier curves across all four CRP–TyG quartiles. **(D)** Landmark Kaplan–Meier analysis excluding malignancies diagnosed within 90 days after the index hospitalization.

Kaplan–Meier analyses were performed to visualize malignancy-free survival according to CRP–TyG strata during follow-up. Overall, patients with higher CRP–TyG levels showed a lower malignancy-free probability over time, consistent with the direction of association observed in the Cox regression models (**Figure 5**).

As shown in **Figure 5A**, the separation between the lowest and highest quartiles emerged early and widened progressively during follow-up, indicating that patients in Q4 experienced a persistently greater cumulative malignancy burden than those in Q1. A similar pattern was observed when the cohort was dichotomized into lower-exposure (Q1–Q2) and higher-exposure (Q3–Q4) groups (**Figure 5B**), supporting the clinical utility of simplified risk stratification.

When all four quartiles were plotted simultaneously, the curves displayed an ordered gradient from Q1 to Q4 (**Figure 5C**). This monotonic separation was consistent with the graded risk increase observed in the primary Cox analyses and further supported the presence of a dose–response relationship across the exposure spectrum.

To address potential reverse causation, a prespecified landmark analysis excluding malignancies diagnosed within 90 days after index hospitalization was also performed. As shown in **Figure 5D**, the overall pattern of lower malignancy-free survival in the higher CRP–TyG strata remained evident after early events were excluded, although the separation between curves was modestly attenuated. This finding indicates that the main association was not solely driven by cancers detected shortly after hospitalization.

### 3.7 Internal validation and clinical utility

To further assess model robustness and the potential clinical utility of incorporating the CRP–TyG composite index into malignancy risk prediction, we performed internal validation using bootstrap resampling and evaluated model performance in terms of discrimination, calibration, and decision benefit.

**Figure 6:**
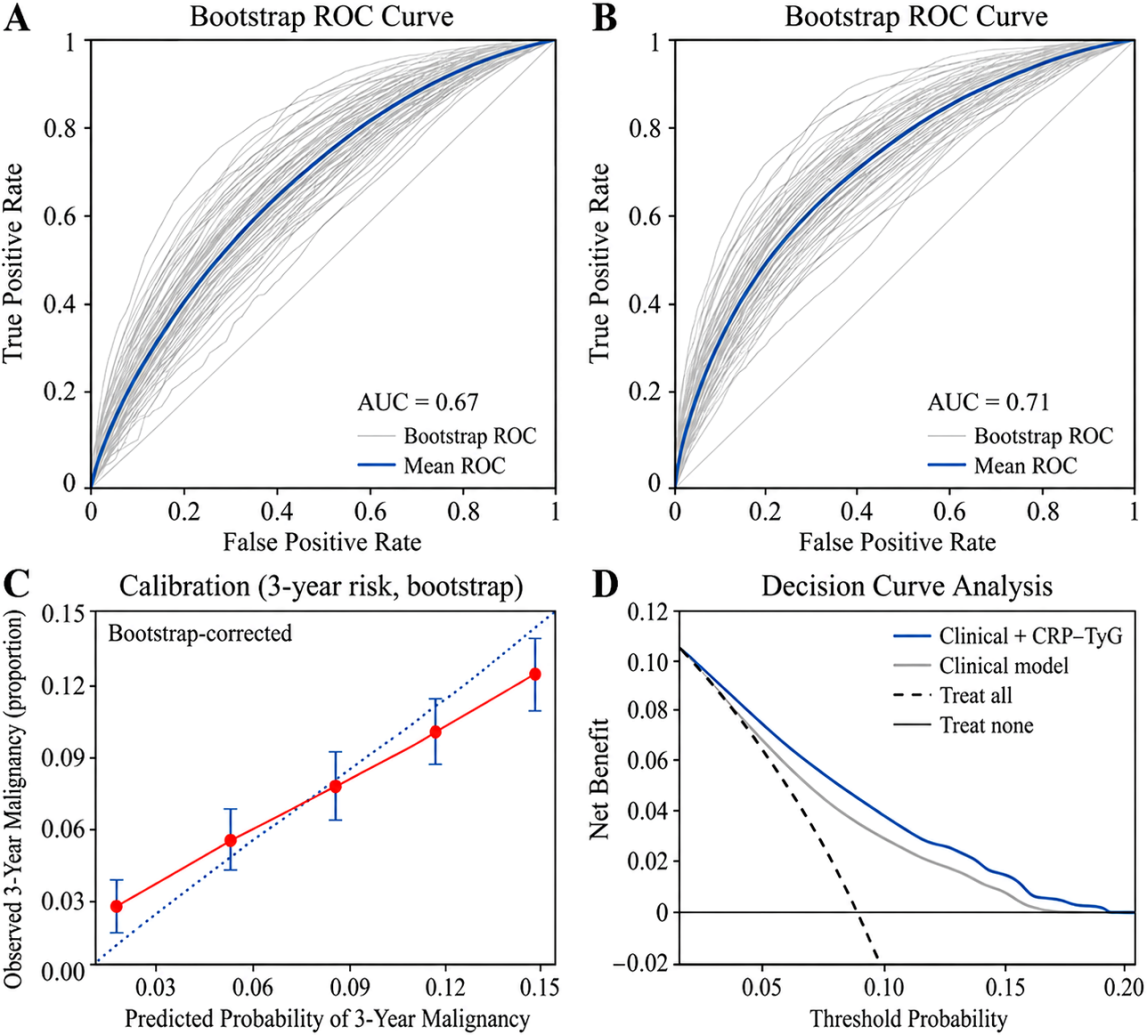
Internal validation and clinical utility of the malignancy risk model incorporating the CRP–TyG composite index. **(A)** Bootstrap receiver operating characteristic curves for the baseline clinical model. **(B)** Bootstrap receiver operating characteristic curves for the clinical model plus CRP–TyG. **(C)** Bootstrap calibration plot for 3-year malignancy risk. **(D)** Decision curve analysis comparing the net benefit of the clinical model, the clinical model plus CRP–TyG, and default intervention strategies.

As shown in **Figure 6A**, the baseline clinical model demonstrated moderate discriminatory ability under repeated bootstrap sampling. The multiple gray curves represent the sampling variability across bootstrap replicates, whereas the blue mean curve represents the average discriminative performance of the model. When the CRP–TyG composite index was added to the clinical model, the average receiver operating characteristic curve shifted outward (**Figure 6B**), indicating improved discrimination. This pattern was consistently observed across bootstrap samples, suggesting that the improvement associated with the composite index was not due to chance fluctuations in a single resampled dataset.

Calibration performance is shown in **Figure 6C**. Overall, the predicted 3-year malignancy risk aligned reasonably well with the observed event rates across the main range of predicted probabilities. Most calibration points were located near the 45-degree reference line, indicating acceptable agreement between predicted and observed risk. Minor deviations were present at the lower-risk end, but these departures were limited and appeared mainly in regions with relatively sparse observations. These findings suggest that the model was not only able to discriminate higher-risk from lower-risk individuals but also provided reasonably accurate absolute risk estimates.

Clinical utility was further evaluated using decision curve analysis (**Figure 6D**). Across a range of clinically relevant threshold probabilities, the clinical model + CRP–TyG generally yielded a higher net benefit than the clinical model alone, as well as the default strategies of intervening in all patients or in no patients. This finding indicates that incorporating the composite index could improve the efficiency of risk-based screening or follow-up strategies by identifying higher-risk individuals more effectively while reducing unnecessary evaluations in lower-risk patients.

Taken together, the bootstrap validation, calibration analysis, and decision curve analysis support the incremental predictive value and potential clinical relevance of adding the CRP–TyG composite index to a conventional clinical risk model.

### 3.8 Subgroup, interaction, and robustness checks

Additional analyses were performed to examine whether the association between the CRP–TyG composite index and incident malignancy remained stable under alternative analytic strategies and across clinically relevant patient subgroups.

Baseline comparisons according to malignancy status are summarized in **Table 5**. Compared with patients who remained free of malignancy, those who developed incident malignancy during follow-up were older, had a longer duration of diabetes, and were more likely to be current smokers and to have comorbid coronary artery disease, heart failure, and chronic kidney disease. They also had less favorable metabolic and inflammatory profiles, including higher HbA1c, fasting glucose, triglycerides, CRP, white blood cell count, neutrophil-to-lymphocyte ratio, TyG index, and CRP–TyG composite score, together with lower HDL-C, albumin, and eGFR. These differences were directionally consistent with the main hypothesis that overlapping inflammatory and metabolic abnormalities characterize the higher-risk phenotype.

**Table 5:** Clinical and laboratory characteristics according to incident malignancy status.

Variable	Malignancy: yes n=344	Malignancy: no n=5,156	P value
Age, years	67.8 ± 10.9	61.7 ± 12.6	<0.001
Male, n (%)	208 (60.5)	2,951 (57.2)	0.228
BMI, kg/m²	26.1 ± 4.0	26.9 ± 4.3	0.001
Diabetes duration, years	11.2 (6.3–16.4)	9.1 (4.8–14.0)	<0.001
Current smoker, n (%)	94 (27.3)	1,012 (19.6)	<0.001
Alcohol use, n (%)	61 (17.7)	736 (14.3)	0.071
Hypertension, n (%)	252 (73.3)	3,538 (68.6)	0.067
CAD, n (%)	112 (32.6)	1,301 (25.2)	0.002
Heart failure, n (%)	58 (16.9)	582 (11.3)	0.001
CKD (eGFR <60), n (%)	139 (40.4)	1,406 (27.3)	<0.001
NAFLD/MAFLD, n (%)	103 (29.9)	1,733 (33.6)	0.154
HbA1c, %	8.3 ± 1.6	7.9 ± 1.7	<0.001
Fasting glucose, mmol/L	9.8 ± 2.9	9.2 ± 3.0	<0.001
Triglycerides, mmol/L	2.05 (1.42–3.02)	1.78 (1.21–2.69)	<0.001
HDL-C, mmol/L	1.03 ± 0.28	1.08 ± 0.31	0.004
LDL-C, mmol/L	2.44 ± 0.77	2.52 ± 0.82	0.062
CRP, mg/L	6.8 (2.9–15.2)	3.9 (1.6–9.4)	<0.001
TyG index	9.28 ± 0.62	8.96 ± 0.66	<0.001
CRP–TyG composite (z-score)	0.68 ± 0.92	-0.05 ± 0.98	<0.001
WBC, ×10^9^/L	7.9 ± 2.5	7.3 ± 2.3	<0.001
NLR	3.1 (2.0–4.7)	2.5 (1.7–3.9)	<0.001
Albumin, g/L	38.1 ± 4.7	40.0 ± 4.6	<0.001
eGFR, mL/min/1.73m²	68.4 ± 24.7	79.2 ± 23.8	<0.001
ALT, U/L	24 (16–37)	26 (17–41)	0.020
Statin use, n (%)	214 (62.2)	2,987 (57.9)	0.108
Metformin use, n (%)	191 (55.5)	3,103 (60.2)	0.074
Insulin use, n (%)	167 (48.5)	2,098 (40.7)	0.004
SGLT2i/GLP-1RA use, n (%)	56 (16.3)	1,006 (19.5)	0.132

**Figure 7:**
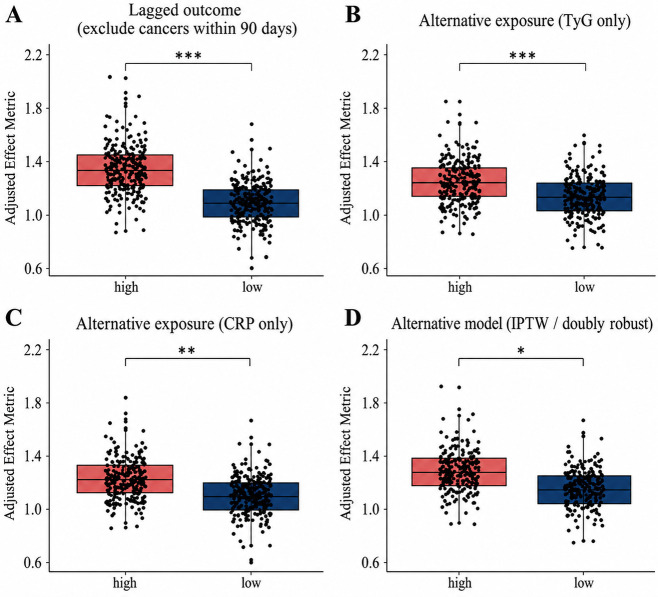
Sensitivity and robustness analyses of the association between the CRP–TyG composite index and incident malignancy. **(A)** Sensitivity analysis after excluding malignancies diagnosed within 90 days after the index hospitalization. **(B)** Sensitivity analysis using TyG-only stratification. **(C)** Sensitivity analysis using CRP-only stratification. **(D)** Sensitivity analysis under alternative model specifications, including inverse probability weighting and doubly robust estimation.

Robustness analyses further supported the stability of the main findings. As illustrated in **Figure 7A**, the association between higher CRP–TyG exposure and malignancy remained evident after excluding malignancies diagnosed shortly after the index hospitalization, arguing against reverse causation as the sole explanation for the observed association. When exposure was alternatively defined using TyG alone or CRP alone (**Figures 7B**, **C**), the direction of association remained positive, but the separation was generally weaker than that observed with the composite index. In addition, analyses using alternative model specifications, including weighted and doubly robust approaches, yielded effect estimates in the same overall direction (**Figure 7D**), indicating that the main conclusion was not materially dependent on a single confounding-control strategy.

Subgroup analyses based on the fully adjusted Cox model are presented in **Table 6**. The association between the highest versus lowest CRP–TyG quartile and incident malignancy was generally consistent across strata defined by sex, age, body mass index, HbA1c, smoking status, statin use, and metformin use. Most interaction tests were not statistically significant, suggesting that the observed association was broadly stable across these clinically relevant subgroups.

**Table 6:** Subgroup and interaction analysis of the association between CRP–TyG and incident malignancy risk.

Subgroup	Stratum (N; events)	HR for Q4 vs Q1 (95% CI)	P for interaction
Sex	Male (3,159; 208)	2.05 (1.40–3.01)	0.312
	Female (2,341; 136)	1.74 (1.08–2.81)	0.312
Age	<65 years (3,080; 143)	1.68 (1.06–2.67)	0.091
	≥65 years (2,420; 201)	2.14 (1.48–3.09)	0.091
BMI	<25 kg/m² (1,915; 129)	2.11 (1.37–3.26)	0.204
	≥25 kg/m² (3,585; 215)	1.79 (1.24–2.60)	0.204
HbA1c	<8.0% (2,624; 141)	1.81 (1.16–2.82)	0.573
	≥8.0% (2,876; 203)	1.98 (1.38–2.84)	0.573
CKD (eGFR <60)	No (3,955; 205)	1.76 (1.20–2.58)	0.028
	Yes (1,545; 139)	2.41 (1.56–3.73)	0.028
CRP level	<3 mg/L (2,204; 92)	1.55 (0.95–2.51)	0.044
	≥3 mg/L (3,296; 252)	2.12 (1.48–3.04)	0.044
Smoking	Never/former (4,394; 250)	1.83 (1.29–2.60)	0.487
	Current (1,106; 94)	2.06 (1.21–3.50)	0.487
Statin use	No (2,299; 130)	2.03 (1.30–3.17)	0.611
	Yes (3,201; 214)	1.85 (1.28–2.67)	0.611
Metformin use	No (2,206; 153)	2.05 (1.36–3.09)	0.418
	Yes (3,294; 191)	1.79 (1.20–2.66)	0.418

Nevertheless, somewhat larger effect estimates were observed in patients with impaired renal function and in those with higher CRP levels. In the subgroup with chronic kidney disease defined by eGFR <60 mL/min/1.73 m², the hazard ratio for Q4 versus Q1 was 2.41 (95% CI 1.56–3.73), compared with 1.76 (95% CI 1.20–2.58) in those without chronic kidney disease (P for interaction = 0.028). A similar pattern was observed across CRP strata, with a stronger association among patients with CRP ≥3 mg/L than among those with lower CRP levels (P for interaction = 0.044). These findings suggest that the relationship between the CRP–TyG composite index and malignancy risk may be more pronounced in patients with greater inflammatory or renal vulnerability, although these interaction findings should be interpreted cautiously.

Overall, the subgroup, interaction, and robustness analyses support the consistency of the primary findings and reinforce the value of the composite index as an indicator of malignancy risk in hospitalized patients with diabetes.

## 4 Discussion

In this retrospective cohort of hospitalized adults with diabetes, we found that the CRP–TyG composite index was independently associated with a higher risk of incident malignancy. The association remained stable after progressive adjustment for demographic factors, metabolic status, organ function, inflammatory markers, comorbidities, and medication use. In addition to the graded increase observed across quartiles, the continuous analysis also supported a positive association, indicating that the relationship was not confined to extreme exposure categories. More importantly, restricted cubic spline and threshold analyses demonstrated that this association was nonlinear, with a clearer acceleration in risk once the composite index exceeded a specific level. Taken together, these findings suggest that the combined inflammatory–metabolic burden captured by the CRP–TyG index may have value for malignancy risk stratification in hospitalized patients with diabetes (10, 11).

A notable finding of this study was that the composite index consistently outperformed CRP or TyG considered separately in discrimination analyses. This result is clinically relevant because it suggests that inflammation and insulin resistance may contribute complementary rather than redundant information. CRP reflects systemic inflammatory activation, whereas the TyG index is a practical surrogate for insulin resistance and metabolic dysregulation. When these two dimensions were integrated into a single metric, the resulting index showed stronger association strength and better discriminatory performance than either component alone. This pattern supports the idea that malignancy risk in diabetes is not adequately characterized by isolated inflammatory or metabolic signals but is more meaningfully captured by their overlap (12, 13).

The nonlinear pattern observed in this study also deserves attention. Rather than increasing in a purely linear fashion across the full exposure range, malignancy risk remained relatively stable at lower CRP–TyG levels and rose more steeply beyond the identified threshold. This suggests that the inflammatory–metabolic burden may become particularly relevant once it reaches a certain level of biological intensity. From a clinical perspective, such a pattern is useful because it implies that the composite index may help identify a subgroup in whom risk escalation is more concentrated, rather than simply indicating a small average increase across the entire population. The threshold finding should not be interpreted as an absolute biological cutoff, but it does provide a practical reference point for future validation and potential risk-based screening strategies (14).

In the site-specific analyses, the strongest association was observed for gastrointestinal cancers, whereas positive trends were also seen for respiratory, urinary, and reproductive system malignancies. These findings should be interpreted cautiously, particularly for cancer categories with fewer events. Nonetheless, the stronger signal for gastrointestinal cancers is plausible in light of the close interplay among metabolic dysregulation, chronic inflammation, gut-associated immune signaling, and digestive system carcinogenesis. At the same time, the generally consistent direction across several organ systems suggests that the CRP–TyG index may reflect a broader host vulnerability rather than a mechanism limited to a single tumor type. Because the number of events was limited in some site-specific analyses, these findings are best regarded as exploratory and in need of confirmation in larger or multicenter datasets.

The subgroup analyses further showed that the association between the CRP–TyG index and malignancy risk was broadly consistent across clinically relevant strata, with most interaction tests being non-significant. This overall consistency strengthens the interpretation that the main association was not restricted to one narrowly defined patient subgroup. At the same time, somewhat larger effect estimates were observed in patients with chronic kidney disease and in those with higher CRP levels at baseline. These patterns may indicate that the index performs particularly well in individuals with greater inflammatory or renal vulnerability, although they should not be overinterpreted. Instead, they are better viewed as signals that may help prioritize future validation work in higher-risk subpopulations (15).

Several biological mechanisms may help explain the observed association. Chronic low-grade inflammation and insulin resistance are both recognized features of the diabetic milieu and have each been linked to carcinogenesis. Insulin resistance may contribute to hyperinsulinemia, activation of insulin and insulin-like growth factor signaling, altered cellular proliferation, and impaired apoptosis. In parallel, sustained inflammatory activation can promote oxidative stress, DNA damage, pro-tumorigenic cytokine signaling, and changes in the tissue microenvironment that favor tumor initiation and progression. The stronger association observed at higher CRP–TyG levels is compatible with the possibility that these processes do not act independently but may reinforce one another once a sufficiently adverse inflammatory–metabolic state is reached. Although our study was not designed to establish mechanism directly, the epidemiologic pattern is biologically coherent.

The clinical relevance of this work lies in the simplicity and accessibility of the composite marker. All components required to calculate the CRP–TyG index are routinely available in standard hospital practice, making it feasible to apply without additional assay burden or specialized testing. In hospitalized patients with diabetes, especially those with multiple comorbidities or high baseline inflammatory burden, such a marker may help support early risk stratification and guide decisions regarding closer surveillance, follow-up intensity, or more individualized screening considerations. Importantly, the landmark and other sensitivity analyses showed that the main association remained directionally stable after excluding malignancies diagnosed shortly after hospitalization, reducing concern that the findings were driven entirely by occult cancers already present at baseline (16).

This interpretation is also consistent with recent oncology studies showing that the TyG index is associated with long-term outcomes in advanced hepatocellular carcinoma, whereas circulating CRP has prognostic relevance in breast cancer across body mass index groups (17, 18). From a methodological perspective, our use of decision curve analysis and bootstrap-based internal validation is aligned with established approaches for evaluating the net benefit and internal stability of prediction models (19, 20). In addition, the reported association between the TyG index and malignant risk in thyroid nodules further supports the broader relevance of insulin-resistance-related markers across malignancy-related clinical settings (21).

This study also has several strengths. It used a relatively large real-world inpatient cohort, applied a fixed baseline exposure window, adopted time-to-event analyses rather than cross-sectional comparisons, and incorporated multiple complementary analytic approaches, including multivariable Cox regression, restricted cubic splines, threshold modeling, competing-risk analysis, internal validation, and decision curve analysis. These features improved the internal coherence of the analysis and allowed the association to be examined from several methodological angles.

Several limitations should also be acknowledged. First, this was a single-center retrospective study, and residual confounding cannot be completely excluded despite extensive adjustment. Second, the CRP–TyG index was derived from a single baseline measurement during hospitalization. Because both inflammatory and metabolic biomarkers may vary over time, some degree of exposure misclassification is possible, which may have attenuated the observed associations. Third, although outcome ascertainment relied primarily on pathology and tumor registry data, some events required supplementary confirmation through diagnostic coding, which may have introduced limited classification error. Fourth, several site-specific cancer analyses were based on relatively small numbers of events, and the corresponding estimates should therefore be interpreted with caution. Finally, because the distributions of CRP, TyG, and related clinical characteristics may differ across populations, the generalizability of our findings to other regions, outpatient settings, or non-hospitalized diabetic populations remains to be established. Future multicenter prospective studies are needed to validate the threshold pattern, test performance across populations, and determine whether longitudinal changes in the CRP–TyG index provide additional prognostic value.

## 5 Conclusion

In conclusion, the CRP–TyG composite index was independently associated with a higher risk of incident malignancy in hospitalized patients with diabetes, and this association followed a clear nonlinear dose–response pattern. Risk increased more markedly once the composite index exceeded the identified threshold, and the association was particularly evident for gastrointestinal cancers, while remaining directionally consistent across subgroup and sensitivity analyses. Compared with CRP or TyG alone, the composite index provided stronger discriminatory performance and added value beyond conventional clinical information. As a simple marker derived from routine laboratory data, the CRP–TyG index may be useful for early malignancy risk stratification in hospitalized diabetic populations. Further multicenter prospective studies are required to confirm its generalizability, refine its clinical threshold, and clarify whether repeated measurement or risk-factor modification can improve long-term cancer prevention strategies.

## Data Availability

The raw data supporting the conclusions of this article will be made available by the authors, without undue reservation.

## References

[1] Yeh HY Pan MH Huang CJ Hong SY Yang HI Yang YY . Triglyceride-glucose index and cancer risk: a prospective cohort study in Taiwan. Diabetol Metab Syndr. (2025) 17:283. doi:10.1186/s13098-025-01768-8. PMID: 40682200 PMC12275392

[2] Zheng X Wang Y Chen Y Liu C Liu T Lin S . Temporal relationship between chronic inflammation and insulin resistance and their combined cumulative effect on cancer risk: a longitudinal cohort study. BMC Public Health. (2025) 25:1501. doi:10.1186/s12889-025-22632-4. PMID: 40269845 PMC12016061

[3] Ruan G-T Shi J-Y Xie H-L Zhang H-Y Zhao H Liu X-Y . Prognostic importance of an indicator related to systemic inflammation and insulin resistance in patients with gastrointestinal cancer: a prospective study. Front Oncol. (2024) 14:1394892. doi:10.3389/fonc.2024.1394892. PMID: 39687883 PMC11646804

[4] Wu Z Yang J Ma Z Chen Y Han M Wu Q . Associations between triglyceride-glucose related indices and the risk of incident pancreatic cancer: a large-scale prospective cohort study in the UK Biobank. BMC Cancer. (2025) 25:327. doi:10.1186/s12885-025-13718-8. PMID: 39984911 PMC11846352

[5] Qiu P Xie J He Y Cong R Huang K Huang B . Association between triglyceride–glucose index and its derivatives and lung adenocarcinoma risk: a case–control study in Chinese adults. Front Endocrinol. (2025) 16:1698373. doi:10.3389/fendo.2025.1698373. PMID: 41427040 PMC12714624

[6] Song C Ping M Lin L Meng X Lan Y Tong H . Association between triglyceride-glucose index and papillary thyroid carcinoma among Chinese adults with thyroid nodules. Front Endocrinol. (2025) 16:1616350. doi:10.3389/fendo.2025.1616350. PMID: 41079184 PMC12507552

[7] Xiao Z Liang Z Chen W Huang H Qu S . Comparative study on the predictive value of TyG, TyG-BMI, and TG/HDL-C for progression-free survival in patients with locally advanced nasopharyngeal carcinoma. Front Nutr. (2025) 12:1657646. doi:10.3389/fnut.2025.1657646. PMID: 41019567 PMC12464962

[8] Liu GM Zhu WB Xu JW . Triglyceride-glucose index predicts postoperative overall survival in hepatocellular carcinoma: a retrospective cohort study. Discov Onc. (2024) 15:651. doi:10.1007/s12672-024-01541-9. PMID: 39537878 PMC11561194

[9] Zhu M Ma Z Zhang X Hang D Yin R Feng J . C-reactive protein and cancer risk: a pan-cancer study of prospective cohort and Mendelian randomization analysis. BMC Med. (2022) 20:301. doi:10.1186/s12916-022-02506-x. PMID: 36117174 PMC9484145

[10] Molodianovitch K Faraggi D Reiser B . Comparing the areas under two correlated ROC curves: parametric and non‐parametric approaches. Biom J. (2006) 48:745–57. doi:10.1002/bimj.200610223. PMID: 17094340

[11] Mondol MH Rahman MS . A comparison of internal validation methods for validating predictive models for binary data with rare events. J Stat Res. (2017) 51:131–44. doi:10.47302/jsr.2017510203

[12] Guo S Zhao Y Jiang Y Ye H Wang Y . Increased pretreatment triglyceride glucose-body mass index associated with poor prognosis in patients with advanced non-small cell lung cancer. Clin Nutr ESPEN. (2024) 59:412–21. doi:10.1016/j.clnesp.2023.12.018. PMID: 38220404

[13] Li F Gao T Li Z Dou H Ba Y Jia S . Triglyceride-glucose index and triglyceride-glucose–body mass index as prognostic factors for early stage breast cancer patients receiving neoadjuvant chemotherapy. Transl Oncol. (2025) 53:102292. doi:10.1016/j.tranon.2025.102292. PMID: 39884219 PMC11814653

[14] Zhu Z Lam TYT Tang RSY Wong SH Lui RNS Ng SSM . Triglyceride-glucose index (TyG index) is associated with a higher risk of colorectal adenoma and multiple adenomas in asymptomatic subjects. PloS One. (2024) 19:e0310526. doi:10.1371/journal.pone.0310526. PMID: 39509387 PMC11542827

[15] Lopez-Ayala P Riley RD Collins GS Zimmermann T . Dealing with continuous variables and modelling non-linear associations in healthcare data: practical guide. BMJ. (2025) 390:1–15. doi:10.1136/bmj-2024-082440. PMID: 40670054

[16] Steyerberg EW Harrell FE Borsboom GJ Eijkemans MJC Vergouwe Y Habbema JDF . Internal validation of predictive models: efficiency of some procedures for logistic regression analysis. J Clin Epidemiol. (2001) 54:774–81. doi:10.1016/s0895-4356(01)00341-9. PMID: 11470385

[17] Li GC Yao ZY Mao HS Han ZX . Association of triglyceride-glucose index with long-term prognosis in advanced hepatocellular carcinoma patients receiving immunotherapy and targeted therapy. World J Gastroenterol. (2025) 31:109863. doi:10.3748/wjg.v31.i30.109863. PMID: 40904882 PMC12404130

[18] Holm JB Baggesen E Cronin-Fenton D Frystyk J Bruun JM Christiansen P . Circulating C-reactive protein levels as a prognostic biomarker in breast cancer across body mass index groups. Sci Rep. (2024) 14:14486. doi:10.1038/s41598-024-64428-3. PMID: 38914635 PMC11196728

[19] Vickers AJ Van Calster B Steyerberg EW . Net benefit approaches to the evaluation of prediction models, molecular markers, and diagnostic tests. BMJ. (2016) 352:i6. doi:10.1136/bmj.i6. PMID: 26810254 PMC4724785

[20] Lankham I Slaughter M . (2020). “ Simple and efficient bootstrap validation of predictive models using SAS/STAT® Software”, in: Proceedings of the SAS Global Forum. Forum, Cary, NC, SAS Institute Inc., 4647–2020.

[21] Qiu X Chen Y Lin R Zhang Z . Association between triglyceride-glucose index and Malignant risk in thyroid nodules: a cross-sectional analysis. J Inflammation Res. (2025) 18:11285–97. doi:10.2147/jir.s533131. PMID: 40860940 PMC12374698

